# A Pilot Study of ^18^F-Alfatide PET/CT Imaging for Detecting Lymph Node Metastases in Patients with Non-Small Cell Lung Cancer

**DOI:** 10.1038/s41598-017-03296-6

**Published:** 2017-06-06

**Authors:** Yue Zhou, Song Gao, Yong Huang, Jinsong Zheng, Yinjun Dong, Baijiang Zhang, Shuqiang Zhao, Hong Lu, Zhibo Liu, Jinming Yu, Shuanghu Yuan

**Affiliations:** 1 School of Medicine and Life Sciences, University of Jinan-Shandong Academy of Medical Sciences, Jinan, Shandong China; 2 Department of Radiation Oncology, Shandong Cancer Hospital Affiliated to Shandong University, Jinan, Shandong China; 3 Shandong Academy of Medical Sciences, Jinan, Shandong China; 4 Shandong Cancer Hospital and Institute, Jinan, Shandong China; 5 Department of Radiology, Shandong Cancer Hospital Affiliated to Shandong University, Jinan, Shandong China; 6 Department of Thoracic Surgery, Shandong Cancer Hospital Affiliated to Shandong University, Jinan, Shandong China; 7 Department of Oncology, Jining Infectious Diseases Hospital, Jining, Shandong China; 8 College of Chemistry and Molecular Engineering, Peking University, Beijing, China

## Abstract

Angiogenesis plays a key role in tumor development and α_v_β_3_ integrin are overexpressed on the endothelial cell surface of newly forming vessels. ^18^F-Alfatide has favorable properties for α_v_β_3_ integrin targeting and showed potential for imaging angiogenesis with Positron Emission Tomography (PET)/computed tomography (CT). In this study, 13 patients with non-small cell lung cancer (NSCLC) who underwent ^18^F-Alfatide PET/CT before surgery were enrolled. The uptake of all dissected lymph nodes (LNs) of ^18^F-Alfatide were assessed visually and analyzed with a maximum and mean standard uptake value (SUV_max_, SUV_mean_) and SUV ratios. LN metastases were pathologically confirmed and 20 of 196 LNs were malignant. All malignant LNs were successfully visualized on ^18^F-Alfatide PET/CT in patients and the sensitivity, specificity and accuracy was 100.0%, 94.9% and 95.4%, respectively. SUV_max_, SUV_mean_ and SUV ratios in malignant LNs were significantly higher than in benign LNs for NSCLC patients (P < 0.001). The same result was observed in patients with adenocarcinoma and squamous cell carcinoma (P < 0.001). The ^18^F-Alfatide parameter shows high sensitivity (83.9–100%), specificity (78.6–96.7%) and accuracy (81.7–96.9%) according to thresholds calculated from receiver operating characteristic curve. Our results suggest that ^18^F-Alfatide PET/CT is valuable in the diagnosis of metastatic LNs for NSCLC patients.

## Introduction

Preoperative staging of mediastinal lymph nodes (MLNs) provides accurate information on the extent of non-small cell lung cancer (NSCLC), it determines the prognosis and guides the choice of therapeutic modalities, consequently is of great importance for patients with NSCLC^1^. As a result, the demand for noninvasive imaging is increased to promote MLN-staging accuracy in NSCLC patients.

^18^F-AlF-NOTA-PRGD_2_ (^18^F-Alfatide), a novel tracer targeting integrin α_v_β_3_, has been studied for angiogenesis imaging by positron emission tomography (PET)^2, 3^. The growth of neovascularization from preexisting ones is called angiogenesis and angiogenesis is a major way in tumor growth and metastases^4^. In the integrin family, one of the most critical molecules involving in tumor angiogenesis and metastases is integrin receptor α_v_β_3_^5, 6^. The integrin α_v_β_3_ which from a class of transmembrane glycoproteins consisting of 18α- and 8β- subunits is researched the most widely and is significantly up-regulated in activated endothelial cells of tumors undergoing angiogenesis, but not expressed in normal cells and quiescent vessel cells^3, 4, 7^. Therefore, tumor angiogenesis can be evaluated by imaging α_v_β_3_ expression, making the integrin receptor α_v_β_3_ a valuable target for diagnosing malignant tumors and metastases^3^.

The various modifications of cyclic arginine-glycine-aspartic acid (RGD) peptides have been labeled with ^99m^Tc^8^ and ^111^In^9^ for SPECT imaging, and with ^18^F^10^, ^64^Cu^11^, ^68^Ga^12, 13^ and ^89^Zr^14^ for PET imaging because integrin α_v_β_3_ can interact with several extracellular matrix (ECM) proteins through the RGD tri-peptide sequence. ^18^F-Alfatide, a new one-step labeled integrin α_v_β_3_-targeting PET probe, is simple and time-saving when synthesized compared with various ^18^F-labelled RGD peptide tracers^3^, including ^18^F-galacto-RGD^15–18^, ^18^F-AH111585^19, 20^, ^18^F-RGD-K5^21^, ^18^FFPRGD2 and ^18^F-FPPRGD2^22^.

In a previous clinical study, this new tracer, ^18^F-Alfatide, is safe when used in the human body and possibly valid in diagnosing primary tumors in patients with NSCLC^3^. Therefore, ^18^F-Alfatide was used as a novel tracer for integrin α_v_β_3_ PET/CT examination in this present research. The objective was to detect lymph node metastases (LNMs) in patients with NSCLC, and to conduct a pilot research of ^18^F-Alfatide PET/CT diagnostic ability in the imaging of mediastinal LNMs in patients with NSCLC.

## Results

After the examination of ^18^F-Alfatide PET/CT, no clinically detectable pharmacologic effects or adverse reactions were observed in any of these subjects. There were no marked changes in laboratory values or vital signs.

**Figure 1:**
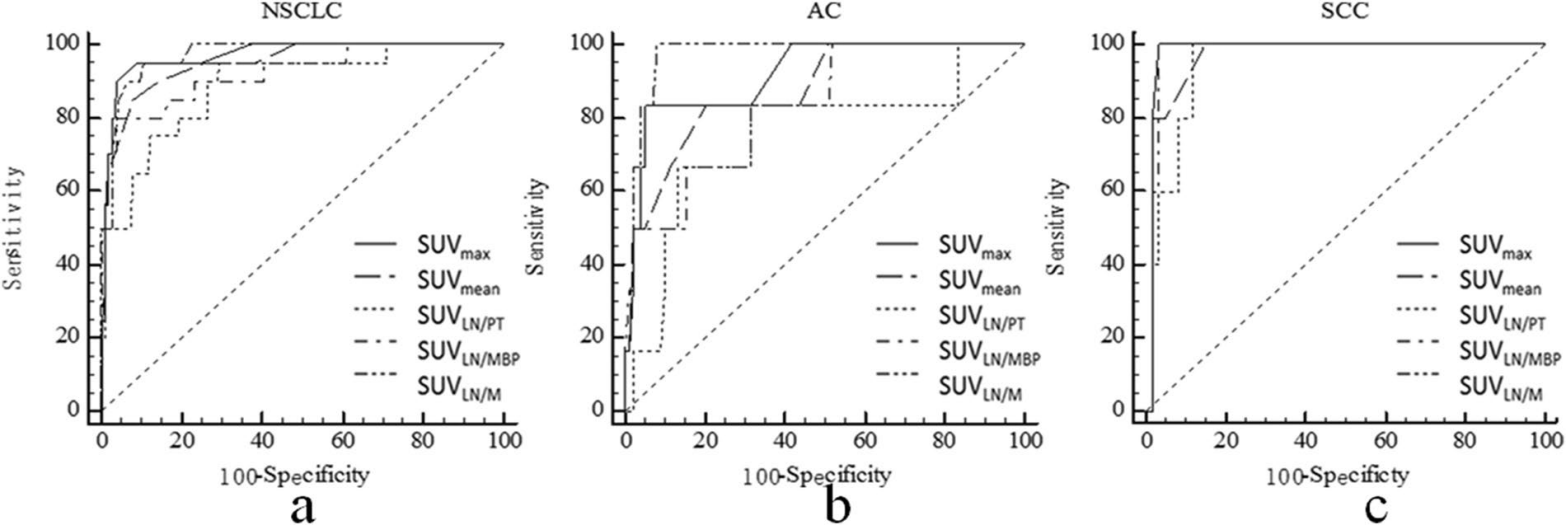
Receiver operating characteristic curves for the semi-quantitative assessment in patients with NSCLC (**a**), adenocarcinoma (**b**) and squamous cell cancer (**c**) at ^18^F-Alfatide PET/CT.

**Figure 2:**
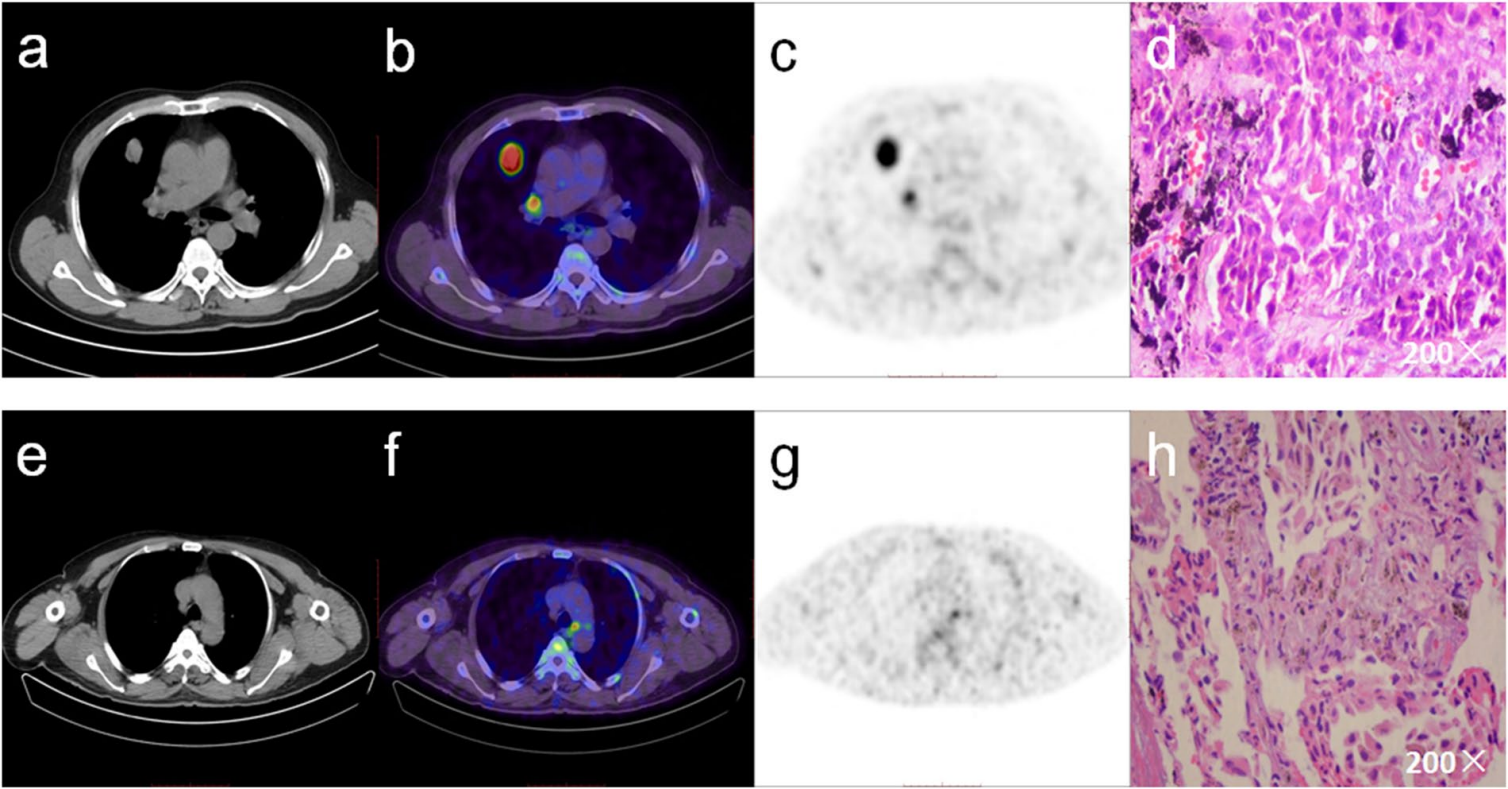
The upper row shows a 62-year-old male suffering from an adenocarcinoma of the upper right lobe. LN station 10 according to Mountain and Dresler^1^ was classified as a true positive case (**a,b,c,d**). The lower row shows a 59-year-old male suffering from a squamous cell carcinoma of the lower left lobe. The increased focal ^18^F-Alfatide uptake in PET/CT imaging is an example of false positive lymph node metastasis (**e,f,g,h**).

Twenty of the resected 196 lymph nodes (10.2%) were malignant in patients with NSCLC, six of 104 lymph nodes were malignant in patients with adenocarcinoma (AC) and 5 of the resected 65 lymph nodes were malignant in patients with squamous cell carcinoma (SCC). All the metastatic lymph nodes could clearly be delineated visually among the resected lymph nodes on the ^18^F-Alfatide PET/CT images. Receiver operating characteristic (ROC) curves for semi-quantitative assessment were illustrated by Fig. 1(a,b,c). Figure 2 the upper row provides an example of lymph node metastasis proved by gold standard and the lower row provides a false positive example.

### Lymph Nodes in NSCLC

#### Visual assessment

Using ^18^F-Alfatide, 20 lymph nodes were correctly recognized in accordance with pathological results and 9 were incorrectly found. Corresponding sensitivity, specificity, negative predictive value (NPV), positive predictive value (PPV) and accuracy were 100.0%, 94.9%, 100.0%, 69.0% and 95.4%.

#### Semi-quantitative assessment

Using ^18^F-Alfatide, malignant lymph nodes (median, 2.15; range, 1.1 to 3.8) had significantly higher maximum standardized uptake value (SUV_max_) (P < 0.001) than benign lymph nodes (median, 0.9; range, 0.3 to 3.3) in patients with NSCLC. The same result was obtained for average SUV (SUV_mean_) in distinguishing between malignant lymph nodes (median, 1.7; range, 0.9 to 2.5) and benign lymph nodes (median, 0.8; range, 0.3 to 2.6; P < 0.001). Table 1 shows statistically significant differences between benign and malignant lymph nodes for SUV_max_, SUV_mean_, and SUV ratios.

**Table 1:** Characteristics of lymph node stations at ^18^F-Alfatide PET/CT.

Variables	NSCLC			AC			SCC		
	Malignant	Benign	P	Malignant	Benign	P	Malignant	Benign	P
SUV_max_	2.5(1.1–3.8)	0.9(0.3–3.3)	<0.01	1.8(1.1–3.8)	0.95(0.3–2.5)	<0.01	1.7(1.4–2.2)	0.9(0.4–3.3)	<0.01
SUV_mean_	1.7(0.9–2.5)	0.8(0.3–2.6)	<0.01	1.45(0.9–2.5)	0.9(0.3–1.8)	<0.01	1.3(1.1–1.8)	0.8(0.4–2.6)	<0.01
SUV_LN/PT_	0.5(0.2–0.9)	0.2(0.1–1.0)	<0.01	0.31(0.2–0.6)	0.24(0.1–1.0)	<0.01	0.3(0.27–0.4)	0.2(0.1–0.6)	<0.01
SUV_LN/MBP_	2.2(0.8–3.5)	0.9(0.3–3.0)	<0.01	1.1(0.8–3.2)	0.8(0.4–2.2)	<0.01	1.9(1.6–2.0)	1.0(0.4–3.0)	<0.01
SUV_LN/M_	4.1(2.2–9.5)	1.7(0.6–4.7)	<0.01	3.0(2.2–9.8)	1.7(0.6–4.2)	<0.01	3.0(2.8–3.4)	1.6(0.8–4.7)	<0.01

All data represent median (range). NSCLC, non-small cell lung cancer; AC, adenocarcinoma; SCC, squamous cell carcinoma; P, P value; SUV, standardized uptake value; LN, lymph node; MBP, mediastinal blood pool; PT, primary tumor; M, muscle.

The optimal cut-off values of SUV_max_ and SUV_mean_ were >1.4 and >1.2 by the ROC analyses (Fig. 1a; Table 2) and their respective areas under the curve (AUCs) were 0.95(95% confidence interval [CI], 0.91 to 0.98) and 0.94 (95% CI, 0.90 to 0.97), respectively. All results are represented in Table 3.

**Table 2:** Receiver operating characteristic analysis: area under the curves and optimal cut-off values.

Parameter	NSCLC			AC			SCC		
	AUC(95%CI)	Cut-off value	P	AUC(95%CI)	Cut-off Value	P	AUC(95%CI)	Cut-off Value	P
SUV_max_	0.95(0.91–0.98)	>1.4	**0.002**	0.86(0.78–0.92)	>1.4	0.02	0.98(0.91–1.0)	>1.3	0.14
SUV_mean_	0.94(0.90–0.97)	>1.2	0.008	0.83(0.75–0.90)	>1.1	0.05	0.97(0.89–1.0)	>1	0.29
SUV_LN/PT_	0.89(0.83–0.93)	>0.3		0.71(0.61–0.79)	>0.3		0.95(0.86–0.99)	>0.3	
SUV_LN/MBP_	0.92(0.87–0.96)	>1.5	0.24	0.82(0.73–0.89)	>0.9	0.40	0.98(0.91–1.0)	>1.5	0.14
SUV_LN/M_	0.97(0.94–0.99)	>2.4	0.01	0.94(0.88–0.98)	>2.4	0.06	0.98(0.91–1.0)	>2.6	0.14

CI, confidence interval; SUV, standardized uptake value; LN, lymph node; MBPS, mediastinal blood pool; PT, primary tumor; M, muscle; P, P value.

**Table 3:** Diagnostic performances of the SUV parameters at ^18^F-Alfatide PET/CT in patients with NSCLC.

NSCLC	Sensitivity		Specificity		PPV		NPV		Accuracy	
	%	95%CI	%	95%CI	%	95%CI	%	95%CI	%	95%CI
Visual	100.0	83.2–100	94.9	90.5–97.6	69.0	49.2–84.7	100	97.8–100	95.4	91.5–97.9
SUV_max_	90.0	68.3–98.2	96.0	92.0–98.4	72.0	50.6–87.93	98.8	95.4–99.9	95.4	91.5–97.9
SUV_mean_	85.0	62.1–96.8	92.1	87.0–95.6	54.8	36.0–72.7	98.2	94.8–99.62	91.3	86.5–94.9
SUV_LN/PT_	85.0	62.1–96.8	81.2	75.3–87.2	34.7	21.7–49.6	98.0	94.2–99.6	82.1	76.1–87.2
SUV_LN/MBP_	85.0	62.1–96.8	96.0	92.0–98.4	70.8	48.9–87.4	98.3	95.0–99.6	94.9	90.8–97.5
SUV_LN/M_	100.0	83.2–100	89.2	83.7–93.4	51.3	34.8–67.6	100	97.7–100	90.3	85.3–94.1

NSCLC, non-small cell lung cancer; LN, lymph node; MBPS, mediastinal blood pool; M, muscle; PPV, positive predictive value; NPV, negative predictive value; CI, confidence interval; SUV, standardized uptake value.

### Subgroups of NSCLC

#### Visual assessment of lymph nodes in AC

11 lymph nodes (10.6%) in patients with AC were recognized visually and six of them (54.5%) were confirmed as pathologically malignant, while five of the 11 lymph nodes (45.6%) were proven to be false positive. Corresponding sensitivity, specificity, NPV, PPV and accuracy were 100.0%, 94.9%, 100.0%, 54.6% and 95.2% (Table 4).

**Table 4:** Diagnostic performances of the SUV parameters at ^18^F-Alfatide PET/CT in patients with AC.

AC	Sensitivity		Specificity		PPV		NPV		Accuracy	
	%	95%CI	%	95%CI	%	95%CI	%	95%CI	%	95%CI
Visual	100.0	54.1–100	94.9	88.5–98.3	54.6	23.4–83.3	100	96.1–100	95.2	89.1–98.4
SUV_max_	83.3	35.9–99.6	94.9	88.5–98.3	50.0	18.7–81.3	98.9	94.2–100	94.2	87.9–97.9
SUV_mean_	100.0	54.1–100	80.6	71.4–87.9	24.0	9.4–45.1	100.0	95.4–100	81.7	73.0–88.6
SUV_LN/PT_	66.7	22.3–95.7	78.6	69.1–86.2	16.0	4.5–36.1	97.5	95.2–99.7	77.9	68.7–85.4
SUV_LN/MBP_	83.3	35.9–99.6	68.4	58.2–77.4	13.9	4.7–29.5	98.5	92.1–100	69.2	59.4–77.9
SUV_LN/M_	100	54.1–100	91.8	84.6–96.4	42.9	17.7–71.1	100	95.4–100	92.3	85.4–96.6

AC, adenocarcinoma; PPV, positive predictive value; NPV, negative predictive value; CI, confidence interval; SUV, standardized uptake value; LN, lymph node; MBPS, mediastinal blood pool; M, muscle.

#### Semi-quantitative assessment of lymph nodes in AC

Using ^18^F-Alfatide, the malignant lymph nodes (median, 1.8; range, 1.1 to 3.8) has significantly higher SUV_max_ (P < 0.001) than in benign lymph nodes (median, 0.95; range, 0.3 to 2.5). The same result was obtained for the SUV_mean_ in distinguishing between malignant lymph nodes (median, 1.45; range, 0.9 to 2.5) and benign lymph nodes (median, 0.9; range, 0.3 to 1.8; P < 0.001). Table 1 shows statistically significant differences between benign and malignant lymph nodes for SUV_max_, SUV_mean_, and SUV ratios.

The optimal cut-off values of SUV_max_ and SUV_mean_ were >1.4 and >1.1 by the ROC analyses (Fig. 1b; Table 2) and their AUCs were 0.86 (95% CI, 0.78 to 0.92) and 0.83 (95% CI, 0.75 to 0.90), respectively. All results are represented in Table 4.

#### Visual assessment of lymph nodes in SCC

9 lymph nodes (13.8%) in patients with SCC were recognized visually and five of them (55.6%) were confirmed as pathologically malignant, while four of the 9 lymph nodes (44.4%) were proven as false positive. Corresponding sensitivity, specificity, PPV, NPV and accuracy were 100.0%, 93.3%, 55.6%, 100.0% and 93.9% (Table 5).

**Table 5:** Diagnostic performances of the SUV parameters at ^18^F-Alfatide PET/CT in patients with SCC.

SCC	Sensitivity		Specificity		PPV		NPV		Accuracy	
	%	95%CI	%	95%CI	%	95%CI	%	95%CI	%	95%CI
Visual	100.0	47.8–100	93.3	83.8–98.2	55.6	21.2–86.3	100.0	93.6–100	93.9	85.0–98.3
SUV_max_	100.0	47.8–100	96.7	88.5–99.6	71.4	29.0–96.3	100.0	93.8–100	96.9	89.3–99.6
SUV_mean_	100.0	47.8–100	85.0	73.4–92.9	35.7	12.8–64.9	100.0	93.0–100	86.2	75.3–93.5
SUV_LN/PT_	60.0	4.6–94.7	91.7	81.6–97.2	37.5	8.5–75.5	96.5	87.9–99.6	89.2	79.1–95.6
SUV_LN/MBP_	100.0	47.8–100	96.7	88.5–99.6	71.4	29.0–96.3	100.0	93.8–100	96.9	89.3–99.6
SUV_LN/M_	100.0	47.8–100	96.7	88.5–99.6	71.4	29.0–96.3	100.0	93.8–100	96.9	89.3–99.6

SCC, squamous cell carcinoma; SUV, standardized uptake value; LN, lymph node; MBPS, mediastinal blood pool; M, muscle; PPV, positive predictive value; NPV, negative predictive value; CI, confidence interval.

#### Semi-quantitative assessment of lymph nodes in SCC

The malignant lymph nodes (median, 1.7; range, 1.4 to 2.2) had significantly higher SUV_max_(P < 0.001) than benign lymph nodes (median, 0.9; range, 0.4 to 3.3). The same result was obtained for SUV_mean_ in distinguishing between malignant lymph nodes (median, 1.3; range, 1.1 to 1.8) and benign lymph nodes (median, 0.8; range, 0.4 to 2.6; P < 0.001).

The optimal cut-off values of SUV_max_ and SUV_mean_ were >1.3 and >1.0 by the ROC analyses (Fig. 1c; Table 2) and their AUCs were 0.98 (95% CI, 0.91 to 1.0) and 0.98 (95% CI, 0.90 to 1.0), respectively. All results are represented in Table 5.

## Discussion

This is a pilot clinical research to evaluate the prognostic value of the ^18^F-Alfatide PET/CT in detecting mediastinal LNMs in patients with NSCLC. This new tracer, ^18^F-Alfatide, has been again proven safe when used in clinical trials, is convenient in the synthesis process, and has shown a significant diagnostic value of mediastinal LNMs.

FDG PET/CT is widely used for detecting LNMs in patients with NSCLC. In a previous meta-analysis, mean sensitivity and specificity of FDG PET/CT for LNMs detection were 69% and 95%^23^. The FDG PET/CT had a relatively high specificity with low sensitivity^24^ and the results were barely satisfactory. Therefore, the researchers focused their attention on the development of new diagnostic methods and new tracers of PET for detecting LNMs in patients with NSCLC. ^11^C-choline^25^, ^18^F-fluorothymidine(^18^F-FLT)^26^, 4′-[methyl-^11^C]-thiothymidine(4DST)^27^, ^18^F-Alfatide are all PET tracers used to visualize various malignancies and LNMs.

In the previous study, the accuracy of ^11^C-choline PET/CT of LNMs detection was 83.76% and the sensitivity was 100%. These results seem to be encouraging, but specificity (72%) was not good enough for diagnosing LNMs in patients with NSCLC^25, 28^. Yamamoto *et al*.^26^ indicated that the diagnostic ability of FLT PET was lower than FDG PET, the sensitivity, specificity, PPV, NPV and accuracy of FLT PET and FDG PET for lymph node staging were 57%, 93%, 67%, 89%, 85 and 57%, 78%, 36%, 91%, 74%, respectively. For 4DST, the results of clinical study suggested that the sensitivity for detecting LNMs was high (82%), but its low specificity (72%) was a limitation (P < 0.001)^27^. Compared with FGD PET/CT and other tracers PET/CT, ^18^F-Alfatide PET/CT showed significantly high sensitivity (85–100%) and specificity (81.2–96.0%) for detecting mediastinal LNMs in patients with NSCLC. The sensitivity, specificity, PPV, NPV and accuracy of ^18^F-Alfatide PET/CT visual analysis were 100%, 94.9%, 69%, 100% and 95.4%, respectively.

Invasive surgical examinations, such as endobronchial ultrasonography transbronchial needle aspiration (EBUS-TBNA)^29^ and mediastinoscopy, showed high specificity and sensitivity for lymph node staging. However, these tests are invasive, and lesion location may restrict the possibility of obtaining tissue samples. ^18^F-Alfatide PET/CT imaging is noninvasive and showed significantly high sensitivity and specificity for detecting mediastinal LNMs in patients with NSCLC. Among all ^18^F-Alfatide PET semi-quantitative parameters, the SUV_max_ showed a better performance and has the potential to serve as the most important semi-quantitative parameter to improve noninvasive nodal staging and treatment planning for LNMs after use of ^18^F-Alfatide PET/CT scans.

Angiogenesis is a vital process in tumor progression and tumor growth, and it is responsible for the metastasis of lymph nodes. Previous work suggests that high expression of integrin α_v_β_3_ on the endothelial cells surface of angiogenesis is related to its proliferative and metastatic properties^30–32^. Therefore, ^18^F-Alfatide is very sensitive to the growth and metastasis of tumors. The high sensitivity of visual and SUV_max_ (Tables 3, 4 and 5) for LNMs indicates that it may be detecting micro node metastases.

In this present study, seven AC cases (54%) and 4 SCC cases (31%) were included, and they showed a similar visual and semi-quantitative analysis outcome as the results from NSCLC patients. Even though the SUV_LN/PT_ was low in both AC and SCC, they did not show significant differences when compared with SUV_mean_, SUV_LN/MBP_ and SUV_LN/M_(P > 0.05). ^18^F-Alfatide PET/CT imaging showed a high sensitivity of SUV_max_ in patients with NSCLC (90.0%) and SCC (100%) but relatively low sensitivity (83.3%) in patients with AC. It is reported that AC is known for low FDG-avidity^33^ and AC may have low affinity with ^18^F-Alfatide as well. This may explain the low sensitivity in AC.

^18^F-Alfatide PET/CT imaging appears to merit LNMs assessment, which is very important for clinical decision-making and surgical planning for NSCLC patients. Even though it has excellent results for lung cancer staging, 9 false-positives occurred in the present study. False-positive uptake was caused by chronic inflammatory and the inflammatory process often accompanying angiogenesis, which produces a large amount of integrin α_v_β_3_^3^. The accuracy (95.4%; 95.2%; 93.9%) of visual analysis was similar with SUV_max_ (95.4%; 94.2%; 96.9%) in patients with NSCLC, AC and SCC. It is also of note that the simpler visual analysis is preferred and SUV would be a secondary aid.

The results indicated that ^18^F-Alfatide PET/CT imaging is potentially successful in diagnosing metastatic lymph nodes with a high sensitivity and specificity in patients with NSCLC. Even though the outcome of this present study is promising, the number of patients was small. Supplementary researches with a larger number of patients would be requested to confirm the results.

## Methods

Thirteen patients including 196 assessable lymph nodes were analyzed (male, n = 6; female, n = 7; median age, 57 years [range, 45 to 69 years]). The including criteria: (1) histologically proven NSCLC, (2) all patients performed lobectomy + lymph node dissection surgery after ^18^F-Alfatide PET/CT imaging, (3) histological results of lymph nodes were available as the gold standard for diagnosis, (4) the age >18 years. Patients histological subtypes included SCC (n = 4), AC (n = 7) and NSCLC not otherwise specified (n = 2). Patient characteristics are represented in Table 6.

**Table 6:** Patients information and diagnosis (n = 13).

Variable	NSCLC (n, %)
**Total**	**13(100)**
**Age(years, range)**	**57(45–69)**
**Gender**	
male	6(46)
female	7(54)
**Histology**	
adenocarcinoma	7(54)
Squamous Cell Carcinoma	4(31)
NSCLC,NOS	2(15)
**N Stage (TNM)**	
0	8(62)
1	2(15)
2	3(23)
3	0(0)

NSCLC, non-small cell lung cancer; AC, adenocarcinoma; SCC, squamous cell carcinoma; NOS, not otherwise specified.

### Approvals

This study was approved by the ethics committee of Shandong Cancer Hospital, and all patients provided written informed consent which included information on radiation exposure. All methods were carried out in accordance with relevant guidelines and regulations.

### ^18^F-Alfatide PET/CT imaging

All patients underwent an ^18^F-Alfatide PET/CT scan using an integrated PET/CT system (Discovery LS; GE Healthcare) from June 2013 to December 2016, which consisted a full-ring dedicated PET of and a spiral CT scan of the same axial range. We purchased PRGD2 peptide that could label with lyophilized kits simply from the Jiangsu Institute of Nuclear Medicine, and the synthesis process was carried out as previous studies^2, 3^. The patients would perform ^18^F-Alfatide PET/CT scan without fasting or receiving CT contrast agents. The radiochemical purity of the ^18^F-Alfatide was higher than 95% after extraction, and its specific radioactivity was higher than 37 GBq (1000 mCi)/μmol. All patients would rest for approximately 60 minutes after injecting ^18^F-Alfatide (212.15 ± 30.8 MBq) intravenously. Axial views were reconstructed into sagittal and coronal views from the top of the neck to the upper abdomen. The patients had normal, shallow respirations during image acquisition. The images were attenuation corrected with the transmission data from CT. The Xeleris workstation (GE Healthcare) viewed the attenuation-corrected PET images, CT images, and fused PET/CT images as coronal, sagittal, and transaxial slices.

### Image analysis

PET data was reconstructed using the ordered-subsets expectation maximization algorithm. The SUV was calculated according to the following formula: (measured activity concentration [Bq/mL] × body weight [g])/injected activity (Bq). Standard visual image interpretation and semi-quantitative analysis were conducted independently by 2 experienced nuclear medicine physicians who were blinded to the clinical and structural imaging findings. All the regions of interest were contoured separately and the SUV_max_ & SUV_mean_ of primary tumor, LNs, aorta and muscle were calculated. The increased ^18^F-Alfatide uptake regions were defined as positive when it showed definite uptake and not related to normal physiologic uptake. And the increased ^18^F-Alfatide uptake area was defined as negative when it was related to the physiologic biodistribution of ^18^F-Alfatide. Care was taken to exclude adjacent blood vessels. SUV ratios of lymph nodes to primary tumor, mediastinal blood pool and muscle were calculated by SUV_LN/PT_, SUV_LN/MBP_, SUV_LN/M_ assessment:

$$\begin{array}{rcl}{{\rm{SUV}}}_{\mathrm{LN}/\mathrm{PT}} & = & {{\rm{SUV}}}_{{\rm{\max }}}(\mathrm{LN}){/\mathrm{SUV}}_{{\rm{\max }}}({\rm{primary}}\,{\rm{tumor}})\\ {{\rm{SUV}}}_{\mathrm{LN}/\mathrm{MBP}} & = & {{\rm{SUV}}}_{{\rm{\max }}}(\mathrm{LN}){/\mathrm{SUV}}_{{\rm{\max }}}({\rm{MBP}})\\ {{\rm{SUV}}}_{\mathrm{LN}/{\rm{M}}} & = & {{\rm{SUV}}}_{{\rm{\max }}}(\mathrm{LN}){/\mathrm{SUV}}_{{\rm{\max }}}({\rm{muscle}})\end{array}$$

### Pathological analysis

All of the patients underwent lobectomy + lymph node dissection surgery. All resected lymph nodes were marked by surgeons during the surgery according to Mountain and Dresler^1^. The tumor specimens were performed according to standard protocols as previously^3^. All hematoxylin-eosin staining tissue sections were reviewed by two pathologists who were blinded to image outcomes and arrived at a single, final conclusion between them. The pathological results were used as gold standard.

### Statistical analysis

Statistical analyses were performed using the MedCalc software (MedCalc^®^, version 15.2.2, 64-bit, MedCalc Software bvba, Ostend, Belgium). All semi-quantitative data are expressed as the median (range). Differences were considered statistically significant when two-tailed P values were less than 0.05. The significant differences between malignant and benign lymph nodes were tested by the Mann-Whitney U test for SUV_max_, SUV_mean_, SUV_LN/PT_, SUV_LN/MBP_ and SUV_LN/M_. The diagnostic performance of SUV parameters in differentiating malignant from benign LNs was analyzed using the ROC curves and AUCs with their 95% CIs. The optimal cut-off values of these variables producing maximum sensitivity plus specificity were determined from ROC analyses. The nonparametric method proposed by DeLong *et al*.^34^ was used to compare correlated ROC curves by MedCalc software.
